# 

**Published:** 1824-06-01

**Authors:** 


### THE MEDICO-CHIRURGICAL REVIEW.

*NEW SERIES.*

VOLUME ONE.

[BEING VOL. V. OF ANALYTICAL SERIES.]

CONDUCTED BY

ASSOCIATED PHYSICIANS AND SURGEONS;

AND SUPERINTENDED BY

JAMES JOHNSON, M.D.

MEMBER OF THE ROYAL COLLEGE OF PHYSICIANS OF LONDON, AND PHYSICIAN EXTRAORDINARY TO HIS ROYAL HIGHNESS THE DUKE OF CLARENCE.

Nec tibi quid liceat sed quid fecisse decebit
Occurrat mentemque domat respectus honesti.—CLAUD.

LONDON:

*Printed by G. Hayden, Little College Street, Westminster.*

Published by Burgess & Hill, Windmill Street, Haymarket; Baldwin, Cradock, and Joy, Pater-Noster Row; Kingsbury, Parbury, & Allen, Leadenhall Street; Highley, and T. & G. Underwood, Fleet Street; Callow & Wilson, Princes Street, Soho; Cox & Son, Borough; Jackson, Borough; Anderson, West Smithfield; John Cox, Berners Street, Oxford Street; Adam Black, Edinburgh; Richard Griffin & Co. Glasgow; Hodges and M'Arthur, Dublin; Messrs. Treuttel & Wurtz, Paris and Strasburg.

1st *JUNE to* 1st *DECEMBER.*

1824.

57440

W1
ME75T

### XIV.

### BIBLIOGRAPHICAL RECORD;

OR,

*Works received for Review since last Quarter.**

☞ *The first five works on the following record came too late last quarter, and were only entered as a postscript in the advertising sheet. They are now regularly entered in the record.*

1. Observations on Vaccination, and on the Practice of Inoculating for the Small-pox. With an Appendix of Cases and Facts. By JOHN CONOLLY, M. D. Physician to the Stratford Dispensary, &c. Octavo, sewed, pp. 76. Price 3s. London, 1824.

2. Nugæ Chirurgicæ; or a Biographical Miscellany, illustrative of a Collection of Professional Portraits. By WILLIAM WADD, Esq. F. L. S. Surgeon Extraordinary to the King, Fellow of the Royal College of Surgeons of London, &c. Octavo, pp. 276. London, 1824.

☞ *Mr. Wadd possesses a very respectable collection of medical portraits; and this work is a kind of catalogue raisonnée of the originals. We shall probably notice some of the biographical sketches in the Review department.*

3. A Course of Lectures on Chemical Science, as delivered at the Surry Institution. By GOLDSWORTHY GURNEY. Octavo, pp. 310, eight plates, price 13s. boards. 1824.

4. A Letter from Goldsworthy Gurney, author of "Lectures on Chemical Science," to W. J. Brande, Esq. Professor of Chemistry at the Royal Institution, on a late Review of Mr. Gurney's Work, in the "Quarterly Journal of Science, &c." Octavo, sewed, pp. 22. 1824. Price 1s. sewed.

☞ *In this Letter Mr. Gurney complains bitterly of the injustice, &c. of a review in Mr. Brande's Journal—but which review he does not impute to the pen of Mr. Brande himself. We must refer to the Journal and the reply for the various* coups *and* contre-coups *which have been given and returned in this literary or rather* scientific *sparring-match.*

*Non nostrum inter vos, &c. &c.*

5. The Animal Kingdom, arranged in conformity with its Organization by the Baron Cuvier, &c. &c. &c. With additional Descriptions of all the Species hitherto named—of many not before noticed; and other original matter By EDWARD GRIFFITH, F. L. S. and others. Octavo, pp. 203, with 18 plates, price 15s.—to be continued quarterly.

☞ *This is the cheapest and most valuable work of the kind in any language. The plates alone are worth the money. Besides the original matter, it will form a complete translation of Cuvier's Work, (4 volumes in the original,) which has never been in an English dress before.*

* NOTE.—Authors and publishers will readily appreciate the importance of having their works recorded, with full length titles, on this list, which stands as a perpetual advertisement so long as the Journal lasts, and as far as it extends. The republication of the Journal in America enhances the advantages of the BIBLIOGRAPHICAL RECORD, on which no work can be entered, unless transmitted, free of expence, to the Editor, under sealed cover to the publishers, or in any other way most convenient to the parties concerned.

6. Elements of Medical Jurisprudence. By Theodoric Romeyn Beck, M. D. Professor of the Institutes of Medicine, &c. &c: in the College of Physicians and Surgeons of the Western District of the State of New York. In two volumes, 8vo. Albany, 1823.

☞ *This is really a very valuable compilation, and does great credit to the judgment and research of the author. We shall notice it frequently in our forensic department.*

---

7. An Enquiry into the Causes of the Curvatures of the Spine, with Suggestions as to the best means of preventing, or, when formed, removing the Lateral Curvature. By T. Jarrold, M. D. 8vo. pp. 147, two plates, London, 1824.

---

8. An Essay on the Blood, comprehending the chief circumstances which influence its Coagulation; the Nature of the Buffy Coat; with a concise Medical View of the State of the Blood in Disease; and an Account of the Powers of a Saturated Solution of Alum as a Styptic Remedy in Hæmorrhage. By Charles Scudamore, M D. F.R.S. Member of the College of Physicians in London, &c. &c. &c. 8vo. pp. 162. London, March, 1824.

---

9. Dissertatio Medica Inauguralis de Rabie Caninâ. By James Lomax Bardsley, M.D. Late President of the Royal Medical Society of Edinburgh; and one of the Physicians to the Manchester Infirmary, &c. Edinburgh, 1823.

☞ *This Essay contains most of what is known on the subject, besides two original cases, with dissections. These last will be noticed in our Periscope.*

---

10. An Attempt to Explain, on Natural Principles, the Cures alleged to be Miraculous, of Miss Lalor and Mrs. Stuart. With an Appendix, containing Cases and Illustrations. By a Physician. 8vo. sewed, pp. 28, Dublin, 1824.

---

11. A Treatise on Syphilis; exhibiting the Advantages of Large Doses of Submuriate of Mercury in the Cure of that Disease. Also an Inquiry into the Modus Operandi of Mercury. Illustrated by Experiments. By James Boyle, Esq. one of the Surgeons to the United Institution of London and Westminster for the Treatment of Lying-in Women and Children; Author of a Treatise on the Epidemic Cholera of India, &c. 8vo. pp. 166, price 6s. boards. London, March, 1824.

---

12. Thoughts on Prison Labour, &c. By a Student of the Inner Temple. 8vo. pp 348. London, 1824.

A Letter on the Nature and Effects of the Tread-Wheel, as an Instrument of Prison Labour and Punishment, addressed to the Right Honorable Robert Peel, M.P. By a Magistrate of the County of Surrey. 8vo. sewed, pp. 74. London, 1824.

A Second Letter to Sir John Cox Hippesley, Bart. on the Mischiefs Incidental to the Tread-Wheel as an Instrument of Prison-discipline: containing an Examination of the Official Reports upon this Subject, returned to the Secretary of State's Office during the present Session of Parliament. By John Mason Good, M.D. F.R.S.

☞ *From the conflicting testimonies that have been published respecting the* Physiology and Pathology *of the Tread-Mill, we cannot come to any positive decision; and we are not much inclined to gather our knowledge, on*

*the present occasion, from* personal experience. *We much doubt whether the Tread-Mill will ever give* entire satisfaction *to the* ladies and gentlemen *who honour it with their patronage. Custom, however, is almost omnipotent. We know what it does for the eels, when skinned alive—and Goldsmith in his Traveller (travellers are allowed some licence) has assured us that—in Switzerland at least—*

"*The rocks* by custom *turn to beds of down.*"

*For our own parts we are much inclined to coincide with Mr. Peel, when he prescribes* change of air *from St. James's Street to Brixton, and change of employment from the Wheel of Fortune to the Tread-Wheel.*

---

13. Symptomatology; or the Art of Detecting Disease: a Lecture occasionally read to the Pupils at the Westminster Hospital, and published according to their request. By A. P. Buchan, M.D. F.L.S. Late Physician to that Institution. To which are added, Tables of Symptoms. 8vo. pp. 190. London, April, 1824.

☞ *The Table of Symptoms is chiefly copied from the symptomatology of Berkenhout, with additions from Hippocrates and others, as well as from the author himself. We shall notice this work shortly, we hope with some approbation.*

---

14. Recherches sur les Propriétés Medicinales et L'Emploi en Medecine de L'Huile de Croton Tiglium, et quelques propositions sur les Maladies de L'Inde. These presentée et Soutenue à la Faculté de Medecine de Paris, le 2d Fevrier, 1824; Par W. E. E. Conwell, de Londonderry (Irlande) Docteur en Medecine de la Faculté de Paris. Membre du College Royal de Chirurgiens à Londres, &c. &c. Quarto, pp. 26. Paris, 1824.

☞ *In this thesis the medicinal properties and best modes of administering croton oil are detailed; but they are now well known in England. At the end of the thesis are four propositions on the diseases of India, occupying only one page. The first proposition is worth insertion here. "In the space of* 15 *years, I have not met with a single case of idiopathic phthisis pulmonalis in India. In that space of time, I observed about seven or eight cases of phthisis occasioned secondarily by affections of the liver.*"

---

15. A System of Anatomical Plates; accompanied with descriptions and Physiological, Pathological, and Surgical Observations. By John Lizars, F.R.S. Fellow of the Royal College of Surgeons, and Lecturer on Anatomy and Physiology, Ed. Part IV. The Muscles of the Trunk. Price 10s. 6d. Plates and Letter-press.

☞ *We anticipated the success of this work, and are happy to find our anticipations fulfilled. We have only to say to the able and indefatigable author*—PERGE PEDE QUO CÆPISTI.

---

16. An Appeal to the Public and to the Legislature, on the Necessity of Affording Dead Bodies to the Schools of Anatomy, by Legislative Enactment. By William Mackenzie, Andersonean Professor of Anatomy and Surgery in the University of Glasgow, &c. 8vo. sewed, pp. 36, Glasgow, 1824.

☞ *This is a well written pamphlet, of which we shall take some notice in another place. We wish this appeal could be made known to the community at large, and to the legislature in particular, in whom the power of remedying the evil essentially resides.*

17. The Apothecaries' Chart; shewing, at one View, the Formulæ of all the Preparations in the London Pharmacpœia of 1824, usually made up by the Apothecary or Retail Druggist:—with Tables of the Weights and Measures; the quantity of Opium, Mercury, &c. contained in Particular Compounds; and a Vocabulary of the Words and Contractions used in the Chart. By a Retail Chemist and Druggist. One large sheet, price 2s. 6d. London 1824.

☞ *This is an extremely useful sheet to hang up in the shop or surgery alongside of Stowe's Toxicological Chart. The author deserves great credit for the ingenious idea.*

---

18. The Chemical Decompositions of the Pharmacopœia Collegii Regalis, Medicorum Londinensis, 1824. To which are added, Tables of the Materia Medica, intended for the Use of Students preparing for their Examinations at Apothecaries' Hall. By JONATHAN PAREIRA, Fellow of the Medical, and Member of the Meteorological Societies of London, Apothecary to the General Dispensary, &c. Octodecimo, pp. 43. Price 1s. 6d.

☞ *We do not know any better way of explaining the nature of this little pocket book than by giving a single specimen—selected more on account of its conciseness than for any other reason.*

"SPIRITUS AMMONIÆ."

"There is here also a double decomposition; Muriate of Potas and Subcarbonate of Ammonia being formed, the latter is volatilised, and dissolved in the spirit." p. 23.

---

19. A Treatise on Stricture of the Urethra; designed as a Manual for the Treatment of that Complaint; and addressed chiefly to Students and Junior Practitioners. By GEORGE MACILWAIN, Member of the Royal College of Surgeons, Surgeon to the Finsbury Dispensary, &c. 8vo. pp. 138, price 6s. boards. London. 1824.

---

20. Lettera del Dottore Giacomo Clark, al Professore Giacomo Tommasini Inlarno alla Letterature Medica Inglese. 8vo. pp. 47. Roma, Decembre, 1823.

---

21. The New York Medical and Physical Journal, Numbers 1 to 5 inclusive, for 1822, and first quarter of 1823. Published quarterly, and edited by Dr. JOHN W. FRANCIS and Dr. JOHN B. BECK, of New York.

☞ *This Journal is on the plan of our respected cotemporary of Edinburgh, though on a much smaller scale, and appears to be conducted in a very respectable and independent manner. We wish the Editors every success. We did not receive Dr. Francis's letter of May 31st,* 1823, *till the 15th April,* 1824. *The following extract from Dr. F's letter will give some idea of the mental activity of our New York brethren.*

"The republication of the MEDICO-CHIRURGICAL REVIEW in this city is "highly encouraged; and when I add that, besides six other American peri- "odical works, on medicine and the collateral branches, arrangements are "making for republishing the MEDICO-CHIRURGICAL TRANSACTIONS, I need "not give you a stronger evidence of the zeal of the medical profession among us."

N. B.—*We have forwarded the last Number of the Analytical Series, through Mr. Millar of New Bridge Street in this metropolis, a liberal and obliging bookseller; and shall transmit to Dr. Francis the new series as it appears.*

22. An Account of the Yellow Fever, as it prevailed in the City of New York, in the Summer and Autumn of 1822. By Peter S. Townsend, M.D. &c. 8vo. pp. 383.

☞ *Dr. Townsend appears to have been a careful observer, and thinks himself authorised by unequivocal facts, to conclude that the fever was imported from the West Indies, and that it was of a decidedly contagious character. Almost the only points on which the physicians of New York agree, are the fatality of the disease, and the little power which any mode of treatment had over it!*

---

23. Section of the Leg, forming part of a Series of Engravings designed as Practical Illustrations of the Surgical Anatomy of the Blood-Vessels, Nerves, &c. relating to Amputation. By Thomas Alcock, Surgeon. One large sheet, with explanations, references, plans, &c. March, 1824.

☞ *This first plate represents, in a most accurate and luminous manner, the face of a stump, when the limb is just severed below the knee. All the muscles, bones, arteries, veins, and nerves, together with the septa between the muscles, are delineated with the greatest fidelity, the parts being of the natural dimensions. We need not say more to recommend it to the student who means to fit himself for the operation in question.*—See Extra-Limites.

---

24. Bulletin des Sciences Medicales. Troisieme Section du Bulletin Universel des Sciences. Publiée sur la Direction de M. Le Baron de Ferussac. Numbers 1, 2, 3, for January, February, and March, 1824. 96 pages each number. (In exchange for Med. Chir. Review.)

☞ *This is a very meritorious production. It is a universal Medical Intelligencer, remarkably well executed.*

---

25. Saggio Sull' Iodio E Sulle differenti sue Combinazione E Preparazioni Farmaceutiche Giusta I Risultamenti che se ne sono ottenuti Nell' Votituto Clinico-Medico dell' J. R. Universitata di Padova. 8vo. pp. 120, Padova, 1822.

☞ *Very creditable to the industry, talent and zeal of the Clinical School of Padua, at the head of which is the illustrious Brera.*

---

26. De Medicis Virtutibus quibus Gaudet Croton Tiglium, ejusque precipue Oleum. Dissertatio inauguralis, &c. Auctore Leo Vita Finzi, ex reparolo Provinciæ Mantuanæ. 8vo. Padua, 1823.

---

27. A Probationary Essay on Cancer; submitted by authority of the President and his Council to the examination of the Royal College of Surgeons of Edinburgh when Candidate for admission into their body. By George Johnston, M.D. Surgeon; Extraordinary Member of the Royal Medical Society, &c. 8vo. pp. 58. Ed. March, 1824.

☞ *This little Essay concentrates most of what is known in the pathology or useful in the treatment of that dreadful disease. Dr. J. exhibits a very fair stock of information on the subject, and acquaintance with the best writers on cancer.*

---

28. The New London Dispensatory, containing a translation of the Pharmacopœia Londinensis of 1824; with the Medical, Natural, and Pharmaceutical History of the Articles in the Materia Medica; the modes of preparing Morphia, Cinchonine, and the other recently discovered Alkaloids; and an explanation of the chemical decompositions, &c. arranged according to a new method. With an appendix, giving an account of Iodine, Hydrocyanic Acid, &c. By Thomas Cox, M.D. *Ed.* &c. 8vo. pp. 352, 10s. 6d. bds. 1824.

☞ *Title page sufficiently explains the scope and tendency of the work.*

---

29. Formulary for the Preparation and Mode of Employing Several New Remedies, &c. by the late Thomas Haden, Esq. *Second Edition,* with numerous alterations and additions. By Robley Dunglison, M.D. &c. 8vo. pp. 150. London, May, 1824.

☞ *This second edition (as will be obvious enough from the position of our second article) reached us long after the review of the first edition was printed off. We have only room to say that Dr. Dunglison has greatly improved the work of his deceased friend. We recommend the publication strongly.*

---

30. The Edinburgh Medical and Surgical Journal, No. 2, New Series for April, 1824, price 6s. (In Exchange for Medico-Chirurgical Review.)

---

31. Medical Report of the Fever Hospital and House of Recovery, Cork Street, Dublin, for the Year 1823. By William Stoker, M.D. Senior Physician to that Hospital, &c. 8vo. pp. 102. Dublin, April, 1824.

---

32. A Short Treatise on the Section of the Prostate Gland in Lithotomy; with an Explanation of a Safe and Easy Method of Conducting the Operation on the Principles of Cheselden. Illustrated by Engravings. By C. Aston Key, Surgeon to Guy's Hospital, and to the Magdalen. 4to. pp. 34, four plates, May, 1824.

☞ *See the surgical division of our Periscope, page* 206, *et. seq.*

---

33. The Surgical Anatomy of the Arteries of the Human Body: designed for the Use of Students in the Dissecting Room. By Robert Harrison, A.B. T.C.D. Member of the Royal College of Surgeons in Ireland and London; and one of the Demonstrators of Anatomy in the School of Surgery in Dublin, &c. In two volumes small octavo. Vol. 1, pp. 226. Dublin and London, May, 1824. Price 5s.

☞ *This little work appears to be one of merit and utility. When we receive the second volume, we shall give some account of the publication to our readers.*

---

34. A Treatise on the Internal Use of the Natural and Factitious Waters of Carlsbad, Marienbad, Ems, &c. By Dr. F. Kreysig, of Dresden, Physician to the King, &c. Translated from the German by Gordon Thomson, M.D. 8vo. pp. 96. Highley, May, 1824.

☞ *Dr. Struve, of Dresden, has established a manufactory of the above-mentioned waters at Brighton, where we hope they will be fairly and liberally tested as to their correspondence with the celebrated native waters on the continent.*

---

35. The Medical and Surgical Register: consisting chiefly of Cases in the New York Hospital. By John Watts, (Jun.) M.D. Valentine Mott, M.D. and Alexander Stevens, M.D. Parts I. and II. 1818, and 1820. With several plates.

☞ *Thanks to the able conductors of the above work.*

36. On the Nature and Symptoms of Cataract, and on the Cure of that Disease, in its Early Stages, by a Mode of Practice calculated to prevent the occurrence of Blindness, and to render unnecessary the Operations of Couching and Extraction. Illustrated by cases. By JOHN STEVENSON, Esq. Fellow of the Royal College of Surgeons, &c. 8vo. pp. 234. London, May, 1824.

---

37. Engravings Illustrative of a Work on the Nature and Treatment of the Distortions to which the Spine and the Bones of the Chest are Subject. By JOHN SHAW, Surgeon, and Lecturer on Anatomy. Folio, pp. 47, of letter-press, 7 plates, and numerous sketches. London, May, 1823.

☞ *We cannot too strongly recommend to our professional brethren the work, of which these plates and descriptions are illustrative. No surgeon should be without the book and plates, who wishes to have a comprehensive and accurate view of the nature, causes, and treatment of spinal diseases, now so prevalent in society. There is a great mass of important matter in the letter-press accompanying these beautiful engravings. We shall revert to the subject in our next number.*

---

38. A Practical Treatise on Diseases of the Skin, comprehending an Account of such Facts as have been recorded on these subjects, with Original Observations. The whole arranged with a View to Illustrate the Constitutional Causes of these Diseases, as well as their Local Characters. By SAMUEL PLUMBE, Member of the Royal College of Surgeons of London, of the Medico-Chirurgical Society, &c. London. Printed for Thomas and George Underwood, 32, Fleet Street. May, 1824.

---

### XV.

### INTELLIGENCE, CORRESPONDENCE, &c.

### *Additional Subscribers since last Quarter.*

B.

Beverley, Dr. St. Petersburgh
Broad, Mr. Charles, Surgeon—To Calcutta

C.

Cannon, Dr. Dublin.
Carrol, Mr. Surgeon
Cordeux, Dr. Ashford, Kent.
Clydesdale Medical Society, Hamilton

D.

Dickinson, Mr. Josiah, Croston, near Ormskirk, Lancashire
Dunn, John, Esq. M. C. S. Hon. Company's Service, Madras

E.

Elliott, Staff Surgeon, Principal Medical Officer, St. Vincent, West Indies

G.

Gaunt, Mr. E. Surgeon, Eccleshale
Gibraltar Medical Society
Greenfield, Mr. Surgeon, White Cross Street, Cripplegate

H.

Hayes, Mr, Surgeon, Upper Charlotte Street, Fitzroy Square
Heinekin, Dr. Madeira.
Hinson, Dr. Henry, Jun. Bermuda.
Hume, Mr. C. Mansfield—To Jamaica
Hume, Dr. John

J.

Johnstone, Mr. William, Surgeon, Calverley
Julius, Dr. Nicol Henry, Hamburgh
Jones, Mr. Surgeon, Portland Town, Regent's Park

M.

Matthews, Dr. Bath
M'Farlane, Dr. J. Glasgow
M'Laren, Dr. His Majesty's Ship Fury, Northern Expedition
Morley, Mr. T. U. Surgeon, Leicester Square

N.

Nicholas, Mr. Charles, Student
Noble, Mr. John, St. George's Hospital
Norwood, Mr. C. Surgeon, Ashford, Kent
Nuttall, Dr. G. R. Russell Place, Fitzroy Square

O.

Ormond, Mr. William, Student
Orr, Dr. Alex. Lochurnoch

P.

Philan, Mr. D. Surgeon, Clonmel, Ireland

R.

Richmond, Mr. Edward, Student, Edinburgh
Roberts, Mr. Member of the Royal College of Surgeons, Dudley
Robertson, Dr. John, Glasgow
Roulston, Mr. Member of the Royal College of Surgeons, Hipperly, Yorkshire

S.

Skaife, Mr. J. Member of the Royal College of Surgeons, Blackburne
Stirling, Alexander, Esq. to India.
Stratford Medical Book Society, Stratford on Avon

T.

Toppe, Mr. Surgeon, Blackburn, Lancashire
Townsend, Dr. Ed. R. Surgeon, Cork

V.

Vaille, Mr. Surgeon, Prospect Place

W.

Waddell, Dr. Calcutta
Wingate, Dr. William

